# The complete chloroplast genome sequence of *Viola verecunda* (Violaceae)

**DOI:** 10.1080/23802359.2021.1997102

**Published:** 2021-11-18

**Authors:** Myounghai Kwak

**Affiliations:** National Institute of Biological Resources, Incheon, Republic of Korea

**Keywords:** Chloroplast genome, *Viola verecunda*, Violaceae

## Abstract

*Viola verecunda* is a perennial plant native to the mountainous areas of Northeast Asia. Here the complete chloroplast genome of *V. verecunda* and its phylogenetic relationships to other species within the genus *Viola* are reported. The complete chloroplast genome of *V. verecunda* is 157,843 bp in length and circular in structure with four regions: a large single-copy region (86,345 bp), a small single-copy region (17,292 bp), and a pair of inverted repeat regions (27,103 bp each). The chloroplast genome contains 111 unique genes comprising 77 protein-coding, 30 unique tRNA, and 4 unique rRNA genes. Based on the protein-coding gene sequences from eight *Viola* chloroplast genomes, with *Balanops balansae* designated as the outgroup, maximum likelihood tree analysis indicates that *V. verecunda* is more closely related to *V. raddeana* than to other *Viola* species. The complete chloroplast genome of *V. verecunda* contributes to a better understanding of the phylogenetic relationships among *Viola* species.

The infrageneric phylogenetic relationships in *Viola* remain poorly understood. Since polyploidization and interspecific hybridization occur very frequently during *Viola* speciation, simple phylogenetic studies using limited numbers of molecular markers fail to provide sufficient resolution to elucidate the complex phylogenetic relationships among *Viola* species (Liang and Xing 2010; Yoo and Jang 2010; Marcussen et al. 2015). Determining the whole chloroplast genomes of *Viola* species may improve the resolution (Cheon et al. 2019). The hidden violet, *Viola verecunda* A. Gray, is commonly found in wet environments of the mountainous areas of Northeast Asia. Species identity of this taxon and delimitation between *V. verecunda* and *V. arcuate* are debated among various researchers (Wang and Huang 1993; Akiyama et al. 2002; Chen et al. 2007; Lee and Yoo 2020). This violet can be distinguished from other *Viola* species by its distinct stems, ovate to cordate leaves, and white flowers. The purpose of this investigation is to assemble and analyze the complete chloroplast genome of *V. verecunda* to contribute to its phylogenetics, systematics and bioinformatics.

*Viola verecunda* plant materials were collected from its natural habitat in Oita Prefecture, Japan (33°8′N, 131°16′E) and the voucher specimen (no. NIBRVP0000736057) was deposited in the herbarium of the National Institute of Biological Resources (KB; www.nibr.go.kr, Myounghai Kwak, mhkwak1@korea.kr). Total genomic DNA was extracted from silica gel-dried leaf samples using the cetyltrimethylammonium bromide (CTAB) method (Doyle and Doyle 1987). Whole-genome sequencing was performed using the Illumina Novaseq 6000 platform (DNA Link Inc., Seoul, Korea). A total of 5.69 Gb raw reads (150 bp paired-end reads) were retrieved and quality-trimmed using the Trimmomatic tool (Bolger et al. 2014). The *de novo* assembly was performed using 36,925,394 (5.51 Gb) reads with GetOrganelle v1.5 software (Jin et al. 2020) and the *V. websteri* chloroplast genome (GenBank accession no. MH229819) as a reference. Annotation of the chloroplast genome was conducted using the GeSeq online program (Tillich et al. 2017) and the annotated genome sequence was deposited in GenBank (accession no. MW586692).

The size of the complete chloroplast genome of *V. verecunda* is 157,843 bp with a GC content of 36.3% and has a typical quadripartite structure. The large single-copy region (LSC; 86,345 bp) and small single-copy region (SSC; 17,292 bp) are separated by a pair of inverted repeats (IRa and IRb; 27,103 bp each). The chloroplast genome of *V. verecunda* contains 111 unique genes comprising 77 protein-coding genes, 30 tRNA genes, and 4 rRNA genes. Among these, 14 genes (*ndhA*, *ndhB*, *petB*, *petD*, *rpl2*, *rpl16*, *rpoC1*, *rps12*, *trnK-UUU*, *trnG-UCC*, *trnL-UAA*, *trnV-UAC*, *trnI-GAU*, and *trnA-UGC*) contained one intron each, and two genes (*ycf3* and *clpP*) contained two introns each, identical with the previously reported *Viola* genomes (Cheon et al. 2019).

To confirm the phylogenetic position of *V. verecunda* within *Viola*, seven complete *Viola* chloroplast genomes were downloaded from GenBank as well as *Balanops balansae* (Balanopaceae) as the outgroup. The nine complete chloroplast sequences were aligned using MAFFT v7.388 software (Katoh and Standley 2013). Phylogenetic trees were calculated using PhyML v3.0 software (Guindon et al. 2010) with a generalized time-reversible (GTR) sequence evolution model and nearest neighbor interchange for tree improvement. Branch support was evaluated with 1,000 bootstrap replicates and the phylogenetic tree was constructed using the maximum-likelihood (ML) method (Figure 1). The phylogenetic positions of the new *V. verecunda* sequences among *Viola* species are shown in Figure 1. *Viola verecunda* was found to be closest to *V. raddeana* in subsection *Bilobatae* under section *Plagiostigma*. In conclusion, the complete chloroplast genome of *V. verecunda* contributes to a better understanding of the phylogenetic relationships among *Viola* species, which will improve genetic diversity assessment and molecular identification within this taxon.

**Figure 1.**
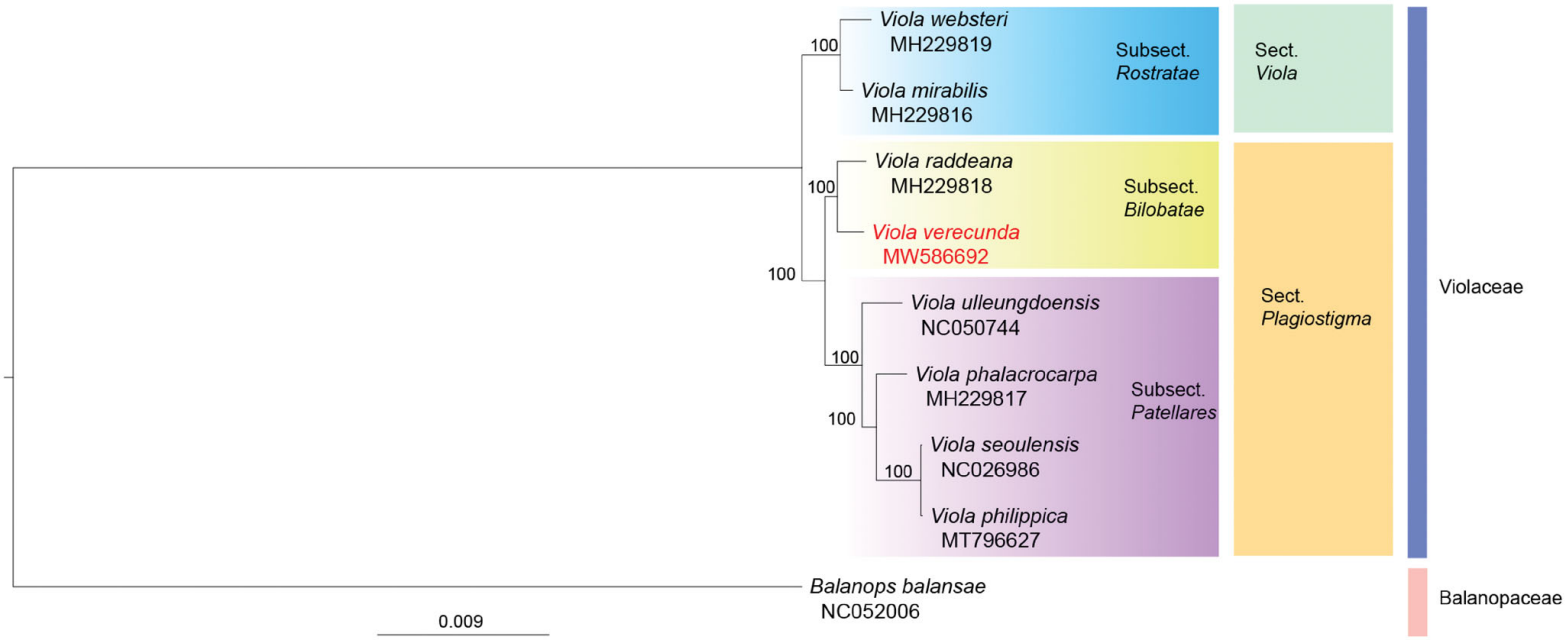
Phylogenetic tree inferred by maximum likelihood using 77 protein-coding gene sequences from the chloroplast genomes of *Viola*, using *Balanops balansae* as an outgroup. The GenBank accession number of each sequence is shown under the species names. Bootstrap support values are displayed on each node.

## Data Availability

The genome sequence data that support the findings of this study are openly available in GenBank of NCBI at (https://www.ncbi.nlm.nih.gov/) under the accession no MW586692. The associated BioProject, SRA, and Bio-Sample numbers are PRJNA742503, SRR15006027, and SAMN19957749, respectively.
